# Molecular Tools for Adapting Viticulture to Climate Change

**DOI:** 10.3389/fpls.2021.633846

**Published:** 2021-02-10

**Authors:** Éric Gomès, Pascale Maillot, Éric Duchêne

**Affiliations:** ^1^EGFV, University of Bordeaux – Bordeaux Sciences-Agro – INRAE, Villenave d’Ornon, France; ^2^SVQV, INRAE – University of Strasbourg, Colmar, France; ^3^University of Haute Alsace, Mulhouse, France

**Keywords:** grapevine, climate change, adaptation, molecular tools, QTL, gene expression

## Abstract

Adaptation of viticulture to climate change includes exploration of new geographical areas, new training systems, new management practices, or new varieties, both for rootstocks and scions. Molecular tools can be defined as molecular approaches used to study DNAs, RNAs, and proteins in all living organisms. We present here the current knowledge about molecular tools and their potential usefulness in three aspects of grapevine adaptation to the ongoing climate change. (i) Molecular tools for understanding grapevine response to environmental stresses. A fine description of the regulation of gene expression is a powerful tool to understand the physiological mechanisms set up by the grapevine to respond to abiotic stress such as high temperatures or drought. The current knowledge on gene expression is continuously evolving with increasing evidence of the role of alternative splicing, small RNAs, long non-coding RNAs, DNA methylation, or chromatin activity. (ii) Genetics and genomics of grapevine stress tolerance. The description of the grapevine genome is more and more precise. The genetic variations among genotypes are now revealed with new technologies with the sequencing of very long DNA molecules. High throughput technologies for DNA sequencing also allow now the genetic characterization at the same time of hundreds of genotypes for thousands of points in the genome, which provides unprecedented datasets for genotype-phenotype associations studies. We review the current knowledge on the genetic determinism of traits for the adaptation to climate change. We focus on quantitative trait loci and molecular markers available for developmental stages, tolerance to water stress/water use efficiency, sugar content, acidity, and secondary metabolism of the berries. (iii) Controlling the genome and its expression to allow breeding of better-adapted genotypes. High-density DNA genotyping can be used to select genotypes with specific interesting alleles but genomic selection is also a powerful method able to take into account the genetic information along the whole genome to predict a phenotype. Modern technologies are also able to generate mutations that are possibly interesting for generating new phenotypes but the most promising one is the direct editing of the genome at a precise location.

## Introduction

### Expected Impacts of Climate Change

The increase of atmospheric CO_2_ concentrations is the main trigger of the greenhouse effect that led to an increase in earth surface temperature (IPCC, 2013). As such, higher (CO_2_) is beneficial to photosynthesis and consequently to plant growth. Indirectly, for an equivalent amount of carbon fixed, an elevated (CO_2_) is associated with higher water use efficiency (WUE), i.e., lower transpiration of water through stomata (Schultz, 2000).

The past increase of temperatures already led to an advance of developmental changes, well documented all over the world. The tight relationship between temperatures and grapevine phenology allows predicting that this trend will continue (Duchêne et al., 2010; Morales-Castilla et al., 2020).

The first consequence of earlier dates of véraison is an increase in temperatures during the ripening period. The ripening period is indeed not only shifting toward the warmest period of summer, at least in the Northern hemisphere, but also temperatures are higher on the same calendar day (Molitor and Junk, 2019). The extent of the advances of budburst dates is still uncertain because they depend on the dates of dormancy release (Leolini et al., 2020), which are difficult to observe and therefore to model. Higher risks of spring frost after budburst should not be overlooked and could increase in vineyards in northern France (Sgubin et al., 2018).

The fulfillment of water needs results from the atmospheric water demand, the soil water availability, and the grapevine canopy architecture. Changes in precipitations in the future are not expected to be uniform, contrasts between wet and dry areas, wet and dry seasons should increase as well as the frequency of extreme precipitation events (IPCC, 2013). The last IPCC report predicts specific regional changes but does not confirm a general tendency of increased drought risks. The evolution of atmospheric water demand is a matter of debate. Using computational methods such as the Penman-Monteith-FAO equation, the evapotranspiration (ET) is believed to increase together with temperatures but trends for a decrease in pan evaporation were also reported (Roderick et al., 2009; Schultz, 2017). The opinion that the water deficit will increase in the future is nevertheless widely shared.

Climate change can have indirect effects on the grapevine by changing the existing equilibrium with pests and diseases. The capacity of the soils to provide nutrients such as nitrogen could also evolve: reduced soil humidity can not only induce water stress but also impair the mineralization of the soil organic matter, and consequently lower nitrogen availability on the top horizons (Curtin et al., 2012).

At last, more frequent extreme events (heavy rains, storms, hail, unexpected cold, or heat waves) can severely impair the long-term sustainability of grape production, but such events are not predictable and technical solutions are difficult to implement.

### Consequences on Yield and Grape and Wine Composition

Climate change can have direct effects on yield components: not only spring frosts can destroy young shoots but higher temperatures around budburst can lower the number of flowers per inflorescence (Petrie and Clingeleffer, 2005). As a consequence of elevated (CO_2_), a higher plant vigor and biomass production in the future, as observed in field-conducted FACE (Free Air Carbon Enrichment) CO_2_ enrichment experiments (Bindi et al., 2005; Wohlfahrt et al., 2018), can likely result in a higher number of inflorescences and flowers per shoot. However, drought during summer can reduce single berry weight in the current season but also lower the number of inflorescences per shoot in the following one (Matthews and Anderson, 1989).

The environmental conditions during ripening concentrate most of the interest, not only of the scientific community but also of producers and winemakers.

Temperatures during ripening are expected to increase, with negative effects on secondary metabolism, such as anthocyanin synthesis (Lecourieux et al., 2017) and faster degradation of malic acid (Lecourieux et al., 2017). Meanwhile, sugar concentrations have increased during the last decades very likely because of the shift of the ripening period toward longer days, and hence, higher global radiation interception. Sugar accumulation could however be limited in the future by reduced water availability, which can not only lower gas exchanges through stomata and photosynthesis activity, but also sometimes impair the ripening process.

Adaptation of viticulture to these changes includes exploration of new geographical areas, new training systems, new management practices, or new varieties, both for rootstocks and scions.

## Molecular Tools for Understanding the Response of Grapevine to Environmental Conditions

Molecular tools can be defined as molecular approaches used to study DNAs, RNAs, and proteins in all living organisms, including grapevine.

A fine description of the regulation of gene expression is a powerful tool to understand the physiological mechanisms set up by the grapevine to respond to abiotic stress such as high temperatures or drought. The current knowledge on gene expression is continuously evolving with increasing evidence of the role of small RNAs, long non-coding RNAs (lncRNAs), DNA methylation or chromatin activity, and, more recently, of alternative transcription of pre-mRNAs.

In parallel, the description of the grapevine genome is more and more precise. After the first release of a whole-genome sequence for the PN40024 line (Jaillon et al., 2007), the genetic variations among genotypes are now revealed by new technologies with very long reads of single DNA molecules (Chin et al., 2016). High throughput technologies for DNA sequencing also now allow the genetic characterization at the same time of hundreds of genotypes for thousands of points in the genome, which provides unprecedented datasets for genotype-phenotype associations studies.

At last, new methods for genome editing open the gate for efficient and stable genetic transformations of the grapevine.

### Transcriptomics

Medium to high throughput transcriptome analysis in grape roots back to the Expressed Sequence Tags (ESTs) programs of the end of the 1990s and the beginning of the years 2000s, which provide the first probe set for the first-generation 3,200 unigenes microarrays used to study grape development (Terrier et al., 2006, 2011). The number of unigenes present on the microarrays rapidly expanded to 14,500 with the Operon (Camps et al., 2010) or Affymetrix (Deluc et al., 2009) grape arrays. Then, with the release of the 12X genome sequence of the PN40024 line, (nearly) genome-wide NimbleGen microarrays, with over 29,000 unigenes represented, were used to study grape transcriptome (Pastore et al., 2014). Full coverage of the grapevine transcriptome was finally achieved by the use of next-generation deep RNA-sequencing (RNA-seq; Zenoni et al., 2010), which provides greater flexibility than microarrays, allowing to work with genotypes distant to the grape reference genome, including non-*vinifera Vitis* species. Both genome-wide microarrays and RNA-seq have been used to characterize the response of grapevine to drought stress (Berdeja et al., 2015; Corso et al., 2015), UV-B/light intensities (Carbonell-Bejerano et al., 2014; du Plessis et al., 2017), and elevated temperature (Rienth et al., 2014, 2016; Lecourieux et al., 2017). Such high-throughput transcriptomics can highlight relevant candidate genes for future breeding programs tailored to produce new grape cultivars better adapted to anticipated climate change conditions, provided that two conditions are met. Firstly, it is paramount that transcriptomics is applied on an eco-physiologically sound and well-characterized experimental plot, with a precise quantitation of the applied stress factor and its physiological impact on the plants (Berdeja et al., 2015). Even the modalities of stress application can be of importance. For example, Rienth et al. (2014) recently demonstrated that the same elevation of temperature applied on grapevine plants during day or night periods led to distinct transcriptomic modulations, suggesting different acclimation responses (Rienth et al., 2014). Secondly, to get the most out of transcriptomic approaches, it is highly recommended to go beyond classical differentially expressed gene analysis and use powerful data mining and meta-analysis tools, such weighted gene co-expression network analysis, that allows identifying co-regulated gene modules and master “switch” genes that are most likely to be key for abiotic stress responses (Palumbo et al., 2014; Hopper et al., 2016; Cochetel et al., 2017). Last, but not least, to the best of our knowledge, all transcriptomic studies published so far on grapevine deal with the response to one single abiotic factor, often applied in controlled or semi-controlled conditions. This is in contradiction with the fact that in the frame of the ongoing global climate change, several abiotic factors will be affected and will most certainly interact to affect grapevine physiology and grape ripening, as evidenced for UV-B and drought (Martinez-Lüscher et al., 2014; Martinez-Luscher et al., 2015a), water availability and elevated temperature (Zarrouk et al., 2016), UV-B, temperature and ambient CO_2_ levels (Martinez-Luscher et al., 2015b; Martinez-Lüscher et al., 2016; Arrizabalaga-Arriazu et al., 2020). Future transcriptomic studies aiming to provide relevant molecular data to breed new cultivars better adapted to future climatic conditions will have to integrate stress combinations in their experimental design.

### Proteomics

Thanks to continuous technological improvement, grapevine proteomic have evolved from 2D gel electrophoresis techniques to large-scale shotgun proteomics using iTRAQ labeling or more recently label-free quantification methods, using multiplexed hybrid mass spectrometers (Vincent et al., 2007; Cramer et al., 2013). Besides transcriptomic, proteomic studies can also provide relevant and complementary information on grapevine response to abiotic stimuli at the molecular level (George and Haynes, 2014; Cramer et al., 2017). Indeed, reports of parallel transcriptome and proteome analysis in response to environmental abiotic factors have shown that transcript levels were not always directly correlated to corresponding protein abundance in various tissues or organs, highlighting the multiple (i.e., transcriptional, translational, and post-translational) levels of gene regulation (Lan et al., 2012; Pan et al., 2012; Wu et al., 2014). This demonstrates the added value of proteomic approaches to decipher grapevine molecular response to climate change-related abiotic factors such as elevated temperature (Liu et al., 2014; Jiang et al., 2017; Lecourieux et al., 2020) or long-term drought stress (Krol and Weidner, 2017).

### Transcriptome Complexification by Alternative Splicing

The full transcriptome includes messenger RNAs (mRNAs) carrying the coding sequences and “non-coding RNAs” (ncRNAs). Recently, alternative splicing (AS) has been shown to participate in the construction of the complete RNA landscape, by being able to generate multiple transcripts from a single multi-exon gene (Reddy et al., 2013). Besides the canonical isoform, a subset of alternative transcripts may arise by intron retention (IR), exon skipping (ES), or usage of alternative splice sites (5′- and 3′-ASS). This notwithstanding, not all alternative transcripts fulfill biological functions, since the use of alternative splice sites may introduce premature termination codons (PTCs) targeting transcripts to the cytoplasmic nonsense-mediated mRNA decay (NMD) pathway (Chaudhary et al., 2019). However, a significant proportion of non-canonical mRNAs are thought to serve in gene expression regulation while some others are likely to encode functional proteins. Like in other plants, AS is ubiquitous in grapevine (Vitulo et al., 2014) and numerous alternative isoforms have been identified and included in the *V. vinifera* reference genome annotation (Canaguier et al., 2017). Both constitutive and AS occur in the nucleus, mainly co-transcriptionally, and are catalyzed by the spliceosome, a macromolecular complex regulated by splicing factors such as serine and arginine-rich (SR) proteins and heterogenous ribonuclear proteins (hnRNPs) (Syed et al., 2012). Interestingly, SR proteins are themselves subjected to differential splicing, notably under stress conditions (Palusa et al., 2007).

Alternative splicing is regulated during plant growth and development, being highly sensitive to environmental signals. Among positive examples, many genes depending on the circadian clock are prone to AS, enabling the plant to rapidly modify its physiological activity in response to changing conditions during the 24-h cycle (Gil and Park, 2019). Light and temperature are the main stimuli modulating the circadian clock: heat stress induces the differential splicing of several core clock genes, the manipulation of which being of particular interest in the view of adaptation to climate warming (Gil and Park, 2019).

**FIGURE 1:**
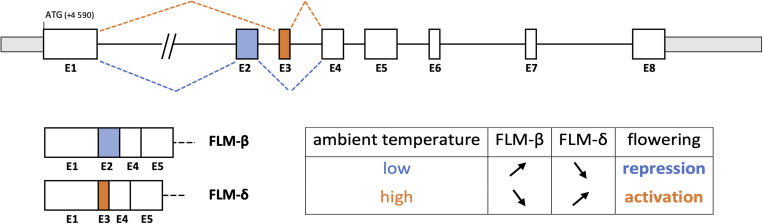
Temperature-dependent alternative splicing of FLOWERING LOCUS M (FLM) in *Arabidopsis thaliana* (Capovilla et al., 2017). Alternative usage of exons 2 and 3, two mutually exclusive exons, acts as a thermosensitive regulator in the flowering time pathway. The FLM-β variant isoform is down-regulated by increasing ambient temperature while the FLM-δ variant is up-regulated, inducing flowering.

A better knowledge of the genetic determinism and AS regulation of phenological traits could also be very helpful for selecting climate-resilient varieties. Precisely, several genes determining the flowering time are submitted to splicing regulation, which modulates their functioning based on light and temperature conditions. For example, the flowering activator CONSTANS (CO) is affected by AS upon light fluctuations, producing a full-size functional protein isoform (COα) and a C-terminally truncated isoform (Coβ) acting as a competitive inhibitor of COα (Park et al., 2019). Moreover, the flowering repressor FLOWERING LOCUS M (FLM) expresses multiple splicing variants, whose predominant isoforms FLM-β (repressor) and FLM-δ (activator) result from alternative usage of two mutually exclusive exons (Nibau et al., 2019). Differential splicing of FLM is controlled by temperature variation, preferentially releasing one or other of these two isoforms for fine-tuning the flowering time (Figure 1).

High light conditions, extreme temperatures, and water stress are powerful inducers of AS, a process therefore supposed to trigger plant adaptation to hard environmental conditions (Filichkin et al., 2018). Heat shock transcription factors (HSFs) conferring heat tolerance, are under the control of the DEHYDRATION-RESPONSIVE ELEMENT BINDING 2 (DREB2) transcription factor, which is differentially spliced in response to abiotic stress, the full-length functional transcript being only produced under stress conditions (Egawa et al., 2006). Moreover, converging evidence suggests that AS modulates the expression of genes of the abscisic acid (ABA) pathway, in response to abiotic stresses (Laloum et al., 2018). One example is provided by the differential splicing of the negative regulator HAB1, a PP2C protein able to dephosphorylate OST1 involved in stomatal movement, leading to the on-off control of the plant response to ABA (Wang et al., 2015). In grapevine, application of a heat shock (35–45°C) greatly modified the leaf transcriptome, AS pattern, and proteome (Jiang et al., 2017). In particular, the transcription level of several SR proteins, as well as their phosphorylation status, a marker of functionality, significantly increased with temperature, showing that the whole splicing machinery was modulated (Jiang et al., 2017; Liu et al., 2019). Because transcription and translation are energy costly, the strong induction of AS under stress conditions is suspected to be a means for reducing the amount and diversity of translatable transcripts (Chaudhary et al., 2019). Intron-retaining transcripts are preferentially produced following abiotic stress application, and accumulate in the nucleus as non-mature isoforms, enabling rapid suspension of translational activity. By this way, nucleus-sequestered transcripts escape to NMD and remain available for further rapid processing and release to the cytoplasm, upon favorable conditions.

Although AS events may be conserved among species and genotypes, some studies have reported on differential AS behavior of distinct genotypes subjected to stress conditions. Two rice varieties, with contrasting levels of tolerance to water stress, showed extensive differential AS when submitted to drought conditions (Zhang and Xiao, 2018). AS divergence affected genes belonging to usual stress response pathways, as well as many spliceosome- and DNA damage repair-related genes that could also be involved in the adaptation to water stress, as suggested by their co-localization with drought-related quantitative trait loci (QTLs) (Zhang and Xiao, 2018). Among others, this strongly suggests that intraspecific genetic variation of components of the splicing machinery itself contributes to differential adaptability to climatic conditions. Similarly, in Arabidopsis, a very low overlap was found between AS patterns of different accessions submitted to temperature changes (Wang X. et al., 2019). DNA polymorphism was associated with AS pattern specificity, most probably accounting for genetic adaptation to distinct native environments. Characterizing genotype-dependent AS patterns in controlled stressful conditions could thus provide an opportunity to identify genes active in stress alleviation. Moreover, the characterization of specific alternative isoforms involved in phenological traits and the response to abiotic stresses should certainly help improving grapevine adaptability to future climate scenarios.

Regulation patterns of transcription intensity and AS, in response to developmental requirements and environmental cues, have most often been reported to overlap poorly, identifying AS as an important process acting independently in transcriptome reprogramming (Karlebach et al., 2020).

### Regulation of Gene Expression: Non-coding RNAs and Micropeptides

There is increasing literature about the role of ncRNAs in the regulation of gene expression patterns in response to environmental conditions, including drought stress (Visentin et al., 2020) and more generally adaptation to climate change (Xu et al., 2019). Small ncRNAs include microRNAs (miRNAs, 21–24 nt) and small interfering RNAs (siRNAs), whereas lncRNAs are RNAs that are more than 200 nt long (Harris et al., 2017) and do not contain an open reading frame. Small RNAs are mobile in the plants and siRNA-dependent epigenetic modifications could be heritable (Pagliarani and Gambino, 2019). RNAs derived from tRNAs and rRNAs also seem to participate in the response to abiotic stress (Cao et al., 2016). siRNAs and lncRNAs also play a role in DNA methylation (Matzke et al., 2015; Tamiru et al., 2018). Additionally, AS is tightly linked to miRNA-mediated regulation of gene expression, in particular via inclusion/exclusion of miRNA target sequences in distinct transcript isoforms, enabling differential regulation by the corresponding small RNA (Yang et al., 2012).

For the grapevine, Belli Kullan et al. (2015) constructed an atlas of miRNAs expression using 70 libraries. They identified 110 already known miRNAs and 185 novel miRNAs. One of their main conclusions is that miRNAs profiling shapes organ identity and that they participate in hormonal regulation. In line with this idea, Carra et al. (2009) had previously identified siRNA 165 as targeting a cytokinin synthase gene, and Wang et al. (2017) VvmiR061 as regulating the gibberellin-signaling pathway. More recently, Rossmann et al. (2020) showed that miR396 participate in the genetic variations of inflorescence architecture in grapevine. Regarding abiotic stress for the grapevine, Leng et al. (2017) showed that miR398 upregulation enhanced the tolerance to oxidative stress and Sun et al. (2015) described the effects of cold on the pattern of miRNAs expression.

**FIGURE 2:**
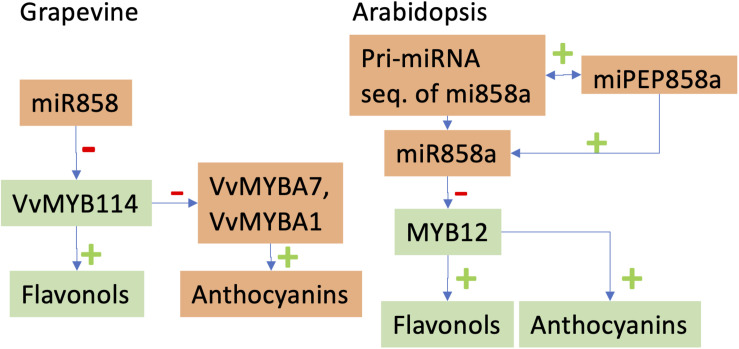
Comparative effects of miR858 in *Vitis vinifera* (Tirumalai et al., 2019) and miR858a in *Arabidopsis thaliana* (Sharma et al., 2020). Levels of elements with different colors vary in opposite directions. In the grapevine, the micro RNA miR858 targets a repressor of the anthocyanin pathway, VvMYB114. In Arabidopsis, the primary miRNA of miR858a encodes for the small peptide miPEP858a. Growing Arabidopsis seedlings in presence of miPEP858a demonstrated that this micropeptide enhances the expression of miR858a.

MicroRNAs profiles are different between irrigated/drought stress conditions but also depend on the grafting combinations (Pagliarani et al., 2017). Pantaleo et al. (2016) also showed the regulations of several miRNAs in response to water stress and to virus infection. In both studies, the expected negative correlation between the abundance of miRNAs and their targeted genes was however not always observed. These results nevertheless open new perspectives for using miRNAs for controlling the genome expression toward a better adaptation to abiotic stress. We can also speculate that miRNAs could be used to control the secondary metabolism of grapevine berries. For example, it was shown that miR828 and miR858 regulate VvMYB114 to promote anthocyanin and flavonol accumulation in grapes (Figure 2; Tirumalai et al., 2019).

Long non-coding RNAs can play a role in the vernalization processes (Liu et al., 2018), in fruit ripening (Arrizabalaga et al., 2018) or in the response to fungal infections (Chen et al., 2018). lncRNAs were identified in the grapevine (Harris et al., 2017; Bhatia et al., 2019; Wang P. et al., 2019) where they participate in many biological functions via interactions with both coding and ncRNAs as well as with transcription factors. They can participate in the response to abiotic stress such as cold stress (Wang P. et al., 2019). To further increase the complexity of gene expression regulation, Chen et al. (2018) also highlighted the role of circular RNAs, related to transposons, in transcriptomic variations in maize leaves.

There is currently no specific knowledge on how to control gene expression in the context of grapevine adaptation to climate change. However, Castro et al. (2016) proved the concept of using miRNAs for genetic engineering by constructing an artificial miRNA precursor, whose corresponding miRNA was able to silent a GFP gene and methods are currently set up for inducing gene silencing by spraying small RNAs on plants (Dalakouras et al., 2016). Application of RNA molecules is even now suggested as a method to trigger RNA interference instead of using genetically modified (GM) organisms (Dalakouras et al., 2020).

Another emerging field is the role of non-conventional micro-peptides in the control of biological processes (Lauressergues et al., 2015; Wang et al., 2020). Regarding the previously cited example of the effects of miR828 and miR858 on anthocyanin and flavonol synthesis in grapevine (Tirumalai et al., 2019), Sharma et al. (2020) demonstrated that pri-miR858a of *Arabidopsis thaliana* encodes a small peptide, miPEP858a, which regulates the expression of miR858a and associated target genes (Figure 2). Chen et al. (2020) also shown that a miRNA-encoded small peptide, miPEP171d1, regulates the formation of adventitious roots. These results increase the complexity of mechanisms of the regulation of gene expression but provide us with tools to better control the phenotypes of grapevine under changing environmental conditions.

### Epigenetics: DNA Methylation and Histone Modifications

The synthesis of an mRNA requires that the corresponding DNA is accessible to the transcriptional machinery. DNA in eukaryotes is wrapped on a structure named chromatin, made of an assembly of proteins called histones. DNA methylation of specific cytosines as well as post-translational modifications (PTMs) of histones, such as acetylation or phosphorylation, determine the accessibility of the genomic information to the transcriptional machinery and the ability to synthesize an mRNA (Gallusci et al., 2017).

DNA methylation and histones PTMs are powerful mechanisms to modulate the gene expression patterns and plant responses to stress (Fortes and Gallusci, 2017). The extent of the actual influence of DNA methylation on gene expression patterns and the level of independence between DNA methylation and genetic variations is however a matter of debate (Seymour and Becker, 2017). Epigenetic changes are part of the developmental program of plants (Gallusci et al., 2017; Shangguan et al., 2020), including sex determination (Latrasse et al., 2017), and can occur in response to changing environments (Fortes and Gallusci, 2017), even at a very small scale (Konate et al., 2020). Epigenetics can be considered as a source of adaptation in perennial species (Brautigam et al., 2013; Gallusci et al., 2017). The heritability and stability of epigenetic changes across generations may however be variable according to the loci (Tricker et al., 2013) or the presence of the initial stress (Tricker et al., 2013). For the grapevine, DNA methylation was shown to participate in the regulation of stilbene synthase genes (Kiselev et al., 2013) and of *VvUFGT*, the gene coding for the anthocyanidin 3-*O*-glucosyltransferase which stabilizes anthocyanidins by glycosylation, allowing red grape varieties to accumulate anthocyanins during maturation (Jia et al., 2020). Histone modifications may also play a role in the regulation of the expression of *VvOMT3*, a gene coding for a methyltransferase (Battilana et al., 2017).

Different methylation patterns were described among grapevine clones of the same variety by methylation-sensitive amplified polymorphism (MSAP) (Ocana et al., 2013). DNA methylation is a dynamic process highly influenced by environmental conditions (Marfil et al., 2019). Methylation patterns (MSAP and methylation-sensitive genotyping by sequencing) in plants of Syrah could be associated with their geographical origin and to the pruning system (Xie et al., 2017). Varela et al. (2020) also showed the effects of the environment on the MSAP profiles but the three clones studied did not respond in the same way, which suggests that epigenetic modifications also depend on genetic variations between clones.

These results raise the idea that environmental conditions can generate clonal variations. For poplar trees, there are indications that clonal history can shape the transcriptomic profiles after modifying the level of DNA methylation (Brautigam et al., 2013).

Recently, using bisulfite sequencing polymerase chain reaction, Jia et al. (2020) demonstrated that the DNA methylation level modulates AS of the *VvDFR* (dihydroflavonol-4-reductase), *VvCHS* (chalcone synthase), and *VvGST* (glutathione-*S*-transferase) genes in ripening Kyoho grapes by IR, altering berry anthocyanin content. Indeed, given the fact that AS proceeds co-transcriptionally, the chromatin state unsurprisingly interferes with splicing regulation (Rahhal and Seto, 2019). For instance, histone acetylation, by inducing chromatin decompaction, speeds up transcription elongation, enabling splicing factors recruitment only at the strongest splice sites and favoring ES. Also, H3K36 methylation, prevalent in actively transcribed gene regions, has been shown to mark genes with temperature-induced AS (Pajoro et al., 2017). It is worth noting that AS could also be implied in stress memories. Priming, which enables the development of a rapid and adequate response to stress after a first exposure, has long been known to be based on heritable chromatin modifications (Mauch-Mani et al., 2017). Interestingly, splicing memory, highlighted by de-repression of AS, has been observed in heat-primed plants after exposure to further lethal stress, suggesting another link between AS and epigenetic footprints (Ling et al., 2018).

If the hypothesis that environmental conditions induce epigenetic adaptations is validated, we can imagine that grapevine plants could be artificially “prepared” for new climatic conditions.

## Genetics and Genomics

### Tools and Methods

The complete sequence of the grapevine genome is available since 2007 after the sequencing and assembly of the nearly homozygous PN40024 line (Jaillon et al., 2007). This first release has been widely used in numerous studies and was improved on the one hand by reducing the number of pseudomolecules representing the chromosomes (Canaguier et al., 2017) and on the other hand by improving the predictions of genes structures, i.e., gene annotations, and the corresponding transcripts. The 12xV2 release of the PN40024 genome^1^ comprises 19 pseudomolecules (for the 19 chromosomes) covering 458,641,822 bp and a pseudomolecule of 2,654,308 bp for all the non-anchored scaffolds. Three sources for gene annotations were used to propose a V3 set of annotations (Canaguier et al., 2017). A total of 42,414 gene structures were predicted but only 15,288 were present in the three annotations sources. Reliable gene annotations are necessary to predict the protein sequences, but also to allow precise quantification of gene expression with RNA sequencing techniques (RNA-seq).

The sequence of the PN40024 line is the reference for identifying genetic variations between genotypes. Resequencing 47 genotypes allowed the design of a DNA chip able to reveal the polymorphisms at the level of a single nucleotide (single nucleotide polymorphism, SNP) at 18,071 positions of the genome. Laucou et al. (2018) used this DNA-chip to characterize 783 different genotypes from the germplasm of Vassal and proposed 118 full parentages and 490 parent-offspring duos. Short reads sequencing was also used to identify variations on the DNA from different clones of Nebbiolo (Gambino et al., 2017) and to characterize progenies by “Genotyping by sequencing” (GBS) (Tello et al., 2019). These high throughput technologies for DNA sequencing give access to a very detailed view of the genetic variability and proved also powerful to identify genes not represented in the reference genome (Da Silva et al., 2013) and to characterize “catastrophic” rearrangements among chromosomes (Carbonell-Bejerano et al., 2017). They however failed to describe the high heterozygosity of the grapevine genome. Single DNA molecule sequencing [Pacific Biosciences^®^ Single-Molecule Real-Time (SMRT) technology] was used for the first time for the Cabernet-Sauvignon genome (Chin et al., 2016). The range of read length was 30–100 kb, giving access to the information on haplotypes, i.e., a precise description of the DNA sequence for each chromosome of the same pair. Gambino et al. (2017) reported that 4,900 new loci could be found in the Cabernet-Sauvignon sequence when compared to PN40024. The Pacific Biosciences^®^ SMRT technology was also used to identify full-length cDNAs in the Cabernet-Sauvignon berry transcriptome, showing the extent of AS (Minio et al., 2019). Recently, a combination of long reads (Pacific Biosciences^®^ SMRT) and short reads (Illumina Hiseq3000 and 2500), allowed the *de novo* phased assembly of the *Vitis riparia* cv. Gloire de Montpellier genome, with a 30× coverage, paving the way for future genome sequence-assisted grapevine rootstock breeding (Girollet et al., 2019).

All these tools and methods are very useful to decipher the links between variations in DNA sequences and traits of interest, especially when considering adaptation to climate change.

### Genetic Determinism of Traits for the Adaptation to Climate Change

Using new varieties or clones is a natural answer when speaking about adaptation to climate change. Present choices of genotypes are adapted to local environmental conditions, soil, meso-climate, microclimate, and to the profile of wine produced. The strategy for local adaptation in the future can be to try to maintain the type of wine that made the renown of the area; it can also consist in a shift, from white to red wines production for example.

If a change in terms of market is possible and accepted, it is likely that technical solutions for adaptation to climate change already exist for most of the grape-growing regions in the world: scion × rootstock × training system combinations are already used for dry and hot environments in the South of Spain, in Chile or Australia.

**FIGURE 3:**
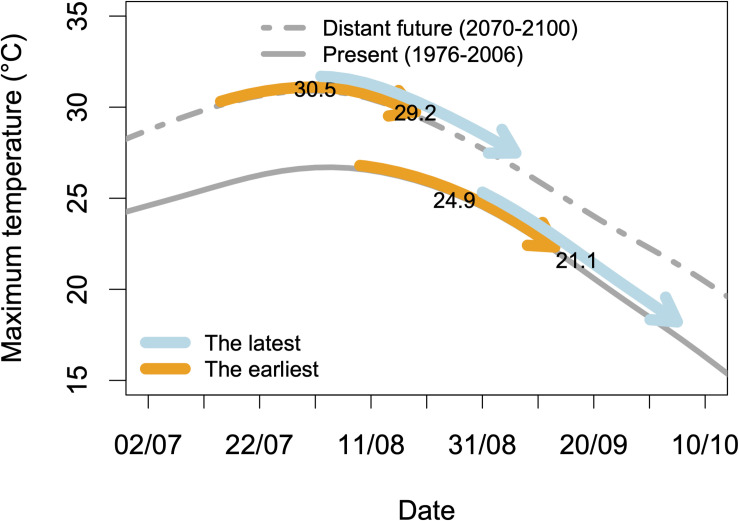
Simulations of maximum temperatures during the ripening period for two virtual extreme genotypes and two climatic datasets. The arrows represent the ripening periods, i.e., 35 days starting at 50% véraison, for two virtual genotypes: the earliest and the latest that should be found in an infinite progeny from a Riesling × Gewurztraminer cross. Two climatic datasets are used: historical data from 1976 to 2006 and simulated data (A1B scenario) for Colmar (48°04′46.3″N 7°21′26.0″E). Details in Duchêne et al. (2010). The figures are the mean values of maximum temperatures during these periods.

The specifications of an ideotype for a variety adapted to climate change can be divided into several chapters. With the aim that the ripening period avoids the warmest periods of summer, a strategy can be to shift this period later in autumn by choosing late genotypes. We could however show that it will be more and more difficult to follow the pace of temperature increase, which shifts the ripening period earlier in summer while the “cool” period moves later in autumn (Duchêne et al., 2010). Another strategy, yet not tested, is to propose very early varieties, whose ripening would take place before the peak of temperatures in summer. In this case, their ripening period would shift toward spring with climate change, in a “self-adaptive” mode (Figure 3). This possibility is however limited by the date of budbreak, which cannot be too early to avoid risks of spring frosts.

The following challenge is also to maintain an economically sustainable yield, especially in the case of drought. New adapted varieties should have a high WUE, i.e., maximize the “crop per drop,” and should be able to maintain the ripening process of the grapes even in case of severe water stress. Keeping an active photosynthetic system under high temperatures or after heatwaves would also be a requested feature but the main challenge is to produce high-quality wines under warm conditions. High temperatures accelerate the degradation of malic acid, impair anthocyanin accumulation, and can be detrimental to aromas or aroma precursors synthesis. The ability to maintain a good acidity of the berries, color, and aromas even under high temperatures is a key expectation for a variety adapted to climate change.

Solutions provided by clonal diversity are the easiest to implement, as they do not require any change in the local legal rules. A lot of accessions are available. In Alsace for example, 1168 clones, representing nine varieties, are present in the INRAE germplasm collections. Contrasted behaviors of Tempranillo clones toward temperatures exist (Arrizabalaga et al., 2018) but the extent of clonal genetic variability useful for the adaptation to climate change might be limited.

Evaluating varieties already cultivated in warm and dry regions is another source of adaptation, but wine producers can be reluctant to adopt varieties previously cultivated elsewhere.

The third way is to create new varieties by breeding. A surprisingly high number of well-renowned cultivars are the progeny of crosses, including Cabernet-Sauvignon, Chardonnay, Merlot, or Syrah (Lacombe et al., 2013). The need for reducing the use of fungicides, but also the idea of adaptation to climate change, has recently stimulated “*de novo*” breeding programs, including in wine-producing areas with protected designation of origin. Molecular markers are key components in these modern approaches.

Whatever the trait of interest, the approach used to detect links between variations in the DNA sequence and values for this trait is the same. First, a population of variable genotypes is requested. It can be extracted from germplasm collections, or created by crossing two varieties (bi-parental cross), or several of them (di-allele cross). The genome of each individual from such a population will be characterized at several loci (points in the genome) by molecular markers. Such markers can be “Simple Sequence Repeat markers” (SSRs), “Single nucleotide polymorphisms,” insertions/deletions (indels), or insertions of retrotransposons. SSRs markers were extensively used for describing the genetic variability within collections (Lacombe et al., 2013), in progeny from crosses (Duchêne et al., 2012), or for clonal identification (Pelsy et al., 2010). SNPs are variations at a single base of the genome. Several methods can be used to characterize the nucleotide present at a precise position of the genome for a given genotype. These methods include direct sequencing of PCR fragments, hybridization on DNA chips, and GBS. GBS is currently one of the most efficient method and can provide thousands of markers for pools of genotypes in a single run (Tello et al., 2019).

Retrotransposons are mobile elements that expand in the genome with a copy paste mechanism and that can also be used as molecular markers (Castro et al., 2012; Villano et al., 2014). One of the most spectacular effects of a retrotransposon is the insertion of Gret1 in the promoter region of a MYB factor that enables the synthesis of anthocyanins. When the insertion is homozygous, berries are white because anthocyanins cannot be synthesized (Kobayashi et al., 2004; Walker et al., 2007).

After the genomic features of the genotypes under study are obtained, the second step is to collect phenotypic information on these genotypes. When crossing two varieties generates the phenotypic variability, mathematical methods for searching loci with a quantitative effect (QTLs) rely on genetic maps that represent the genetic links between loci. The thousands of grapevine genotypes available are another source of variability. Because it is not possible to study at the same time all of them, specific panels, designed for association studies, are constituted (Nicolas et al., 2016). Using dense information on DNA variations among individuals from these panels, “Genome-wide association studies” (GWAS) can search for relationships between genomic and phenotypic data, locus by locus (Nicolas et al., 2016; Guo et al., 2019; Liang et al., 2019). Finally, “genomic selection” methods try to fit mathematical models that use all the genetic information available to predict the value of a trait (Meuwissen et al., 2001; Fodor et al., 2014).

### Molecular Markers for Developmental Stages

Quantitative trait locus detection was performed on several progenies and yielded several QTLs for budburst, flowering, and veraison. QTLs for budburst are rare (Duchene et al., 2012) and are difficult to detect because budbreak is the consequence of two phenomena: the date of dormancy release and the heat requirements between this date and actual leaf appearance. Table 1 summarizes the QTLs detected for flowering time and veraison, including with GWAS (Laucou et al., 2018). Using the same type of data, Delfino et al. (2019) identified four veraison meta-QTLs located on linkage groups 1 and 2, and additional meta-QTLs on LG 14, 16, and 18.

**TABLE 1:** Main QTLs for developmental stages.

Chromosome	Flowering time or budbreak-flowering duration	Date of véraison, flowering-véraison duration, or budburst-véraison duration
1	Costantini et al., 2008; Fechter et al., 2014	Fechter et al., 2014
2	Costantini et al., 2008; Grzeskowiak et al., 2013	Costantini et al., 2008; Grzeskowiak et al., 2013
3		Laucou et al., 2018
4	Fechter et al., 2014	
6	Costantini et al., 2008	Costantini et al., 2008
7	Duchene et al., 2012; Grzeskowiak et al., 2013	
8	Fechter et al., 2014	
11	Fechter et al., 2014	Fechter et al., 2014
13		Fechter et al., 2014
14	Duchene et al., 2012; Fechter et al., 2014	
15		Grzeskowiak et al., 2013
16	Fechter et al., 2014	Duchene et al., 2012; Zyprian et al., 2016; Costantini et al., 2008
17	Fechter et al., 2014	Grzeskowiak et al., 2013
18		Duchêne et al., 2012; Zyprian et al., 2016
19	Fechter et al., 2014	

The results from QTL studies show that it is possible to find some genetic explanations for the high range of phenological stages among grapevine varieties (Parker et al., 2013; Laucou et al., 2018). By combining specific alleles, it is possible to imagine and to try to create new genotypes with desired features (early or late véraison for example). Such genotypes are called “ideotypes.” Regarding adaptation to climate change, new genotypes created today will be cultivated 15–20 years ahead under different environmental conditions. Only a few traits, such as resistance to diseases or berry color, are stable under a changing environment. To predict behavior in the future, a modeling step is necessary. Mechanistic models can predict phenotypic values using environmental variables and genetic specific model parameters. This approach was used for maize (Reymond et al., 2003), peach (Quilot-Turion et al., 2016), tomato (Prudent et al., 2011), and cauliflower (Rosen et al., 2018).

Duchêne et al. (2010) provided an example of such an approach for the developmental stages of the grapevine. The use of heat summations between 15 February and budbreak, budbreak and flowering and flowering to véraison proved efficient to predict the dates of budbreak, flowering, and véraison for Riesling and Gewurztraminer (Duchêne et al., 2010). This model was used to give an estimate of the advance of phenological stages in the future. In a second step, independent QTLs were identified in the progeny of a Riesling × Gewurztraminer cross for the parameters of this model for grapevine phenology (Duchene et al., 2012). This allowed the construction of virtual genotypes: the earliest and the latest one that could be found in an infinite progeny by combining in a single genotype, on the one hand, all the alleles shortening the different phases, and on the other hand all the alleles with the opposite effects. Such virtual genotypes can be projected in the climate of the future and their interest compared (Figure 3). This result would not have been possible without molecular markers and the identification of QTLs. Moreover, breeding desired genotypes with marker-assisted selection (MAS) will use the same molecular information.

### Molecular Markers for Water Use Efficiency

Increasing water stress is a major concern in the adaptation of viticulture to climate change. There is a large genetic variability of the responses to water shortage both for scions (Tomás et al., 2014) and rootstocks varieties (Serra et al., 2014 for a review).

Many traits and mechanisms are involved in the response of a rootstock × scion combination to the water demand/water availability ratio.

Considering rootstocks, they can differ by their capacity to extract water from the soil, which is primary linked to root biomass, but also to the hydraulic conductivity of the roots. The stomatal aperture is under the control of ABA, which is mainly synthesized by the roots in response to drought. ABA could also partly control the hydraulic conductance of the leaves (Simonneau et al., 2017). The genes responsible for the genetic variations of these traits are not yet precisely identified but the information provided by molecular markers is increasingly affordable.

**FIGURE 4:**
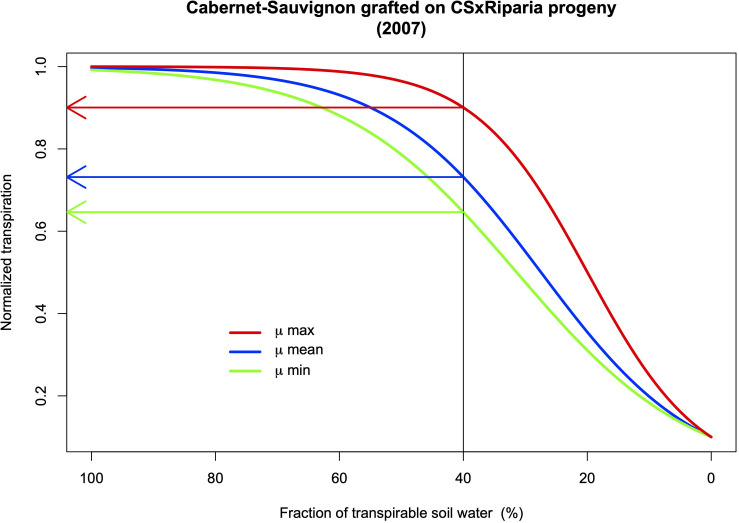
Simulations of scion normalized transpiration rate (NTR) for Cabernet-Sauvignon according to rootstock genotypes in response to the fraction of soil transpirable water (FTSW). The relationship was: NTR = 1/(1 + 9 × e^–μ × FTSW^). μ values calculated for 2009 (Marguerit et al., 2012). A QTL on chromosome 13 was identified for the μ parameter.

Tandonnet et al. (2018) measured seven traits related to root architecture in the vineyard in the progeny of a Cabernet-Sauvignon × Riparia Gloire cross used as rootstocks for five scion varieties. They identified several significant QTLs on chromosomes 1, 2, and 5 for root biomass for example. Interestingly, a QTL for aerial biomass and QTLs for the aerial: root ratio were detected on different chromosomes (3 for the first trait; 6, 9, and 18 for the second). This means that it is likely possible to breed rootstocks with high root biomass, and a good water extraction capacity, while controlling aerial growth, the evaporative surface, and consequently water demand. The link between the response to drought stress and root/aerial biomass was not established in this study, but using the same progeny in a drought stress experiment with potted plants, Marguerit et al. (2012) identified several QTLs from the rootstock that control the transpiration rate by the scions. They also detected a QTL for a coefficient for the mathematical relationship between the changes in soil water availability and the transpiration rates (Figure 4) that can be integrated into modeling simulation of ideotypes of rootstocks.

These results show that the control of the response to water stress depends on many genes from the rootstock and that the combination of alleles for the “ideal” rootstock adapted to drought is not straightforward. It however shows which traits are inter-dependent which is essential for preparing future studies but also for identifying targets for breeding programs.

The response of the scion to drought depends on the roots but genetic studies highlighted the complexity of the components of the aerial part. The study under well-watered and moderate stress conditions of the progeny from a Syrah × Grenache cross grown in pots on a phenotyping platform provided key results. Coupel-Ledru et al. (2014) identified in this experiment QTLs for leaf area, specific transpiration rate, specific hydraulic conductance, or minimal daytime leaf water potential. These QTLs, spread over 10 chromosomes, were partly independent, showing that global behavior depends on many factors under genetic control. The same progeny was also used to demonstrate that nighttime transpiration was a major component of the genetic variability (Coupel-Ledru et al., 2016). Nighttime transpiration was partly due to incomplete stomatal closure at night (estimated to 70%) and to water loss through the cuticle (estimated to 30%). A genetic variability exists for both components. Stable QTLs for nighttime transpiration were identified on chromosomes 1, 4, and 13. More importantly, these QTLs did not colocalize with QTLs for daytime transpiration. This means that is possible to partly uncouple the overall capacity of photosynthesis (correlated to daytime transpiration) to overall water losses, which opens new perspectives to breeding programs. The availability of molecular tools for genetic studies was pivotal in this approach.

### Molecular Markers for Stable Berry Quality

Possible effects on grape characteristics and modifications of the aroma profiles are the main concerns about climate change.

**FIGURE 5:**
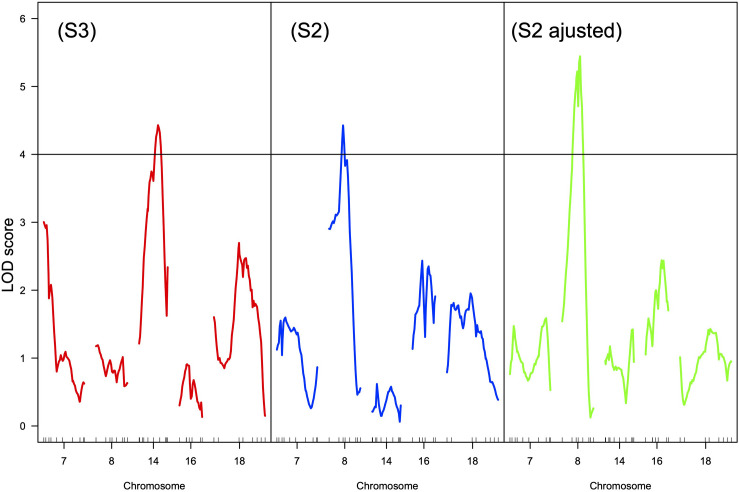
LOD curves for the mean sugar content of the berries over 3 years in progeny from a Riesling × Gewurztraminer cross according to three sampling procedures: (S3) at harvest, same date for all the genotypes, (S2) 230 degree.days (base 10) after mid-véraison, (S2 adjusted) 230 degree.days (base 10) after mid-véraison but adjusted for the fruit to leaf ratio (Duchêne, 2015). The horizontal line is the genome wide LOD threshold at *p* = 0.05. Small vertical ticks on the *X*-axis represent the position of molecular markers on the genetic map. Chromosomes 7, 14, 16, and 18 are presented because QTLs for development stages were detected (Duchene et al., 2012).

Increasing sugar content currently leads to high alcoholic contents of the wines, reducing their drinkability (Alston et al., 2011) and the consumers’ willingness to pay (Tempere et al., 2019). The decoupling between sugar accumulation and anthocyanins synthesis is also a major concern (Martinez de Toda et al., 2014). For a given genotype, the final sugar content of the grape berries is determined by the leaf to fruit ratio (Duchêne et al., 2012) and by the photosynthetic conditions during ripening (solar radiation temperature, water availability, …). Training systems and vineyard geographical position, as well as genetic diversity, can help to counterbalance the expected increase of sugar accumulation (van Leeuwen et al., 2019). The range of genetic variability for sugar content in germplasm collections, measured as total soluble contents (TSS in °Brix), can indeed reach 13.7–31.5°Brix (678–1784 mmol.L^–1^ sugars) between different cultivars (Kliewer et al., 1967; Liu et al., 2006). It is however clear that the way the sampling date is chosen can have undesirable effects on the evaluation of genetic effects (Duchêne et al., 2012). To overcome this difficulty Bigard et al. (2018) proposed to collect berry samples when berry volume reaches a maximum, i.e., when phloem uploading ceases. They recorded variations from 813 to 1353 mmol.L^–1^ of sugars among *V. vinifera* varieties, which confirms the reality of a genetic variability for sugar accumulation capacities at a precise physiological stage. QTLs for sugar content were described in different segregating progenies but their effects were weak (Chen et al., 2015; Houel et al., 2015) or observed only during one season (Yang et al., 2016). Ban et al. (2016) identified a QTL for TSS on chromosome 2 that explained more than 20% of the phenotypic variance over two seasons. However, TSS was significantly negatively correlated to harvest dates and the QTL detected might result from confusing effects. The data published on QTLs for sugar accumulation did not distinguish between the role of developmental stages, fruit load, and leaf area. Duchêne et al. (2012) demonstrated that the variability of TSS measured on the same date in progeny from a cross between Riesling and Gewurztraminer was mainly explained by the dates of véraison and by the fruit to leaf ratio. By collecting berry samples after the same heat summation after the onset of ripening for each genotype and by correcting the measured values according to the fruit to leaf ratio, a QTL on chromosome 8 can be detected (Figure 5) whereas the likelihood of a QTL on chromosome 14, where was previously detected a QTL for flowering time (Duchene et al., 2012), is no longer significant. The allelic effect at the locus on chromosome 8 represents approximately 1°Brix, i.e., 0.7% v/v potential alcohol. This is not negligible but building ideotypes for controlling sugar accumulation taking into account the yield potential, the leaf area (plant vigor), the earliness at véraison and a supplementary QTL more closely linked to berry physiology might take too much effort when compared to changing training systems and management practices such as leaf removal.

Exploring a genetic context beyond the unique *V. vinifera* species can open new perspectives: some progenies from species such as *Vitis rotundifolia* exhibit a low ability to accumulate sugars (Salmon et al., 2018) and the underlying genetic architecture is under study (Torregrosa et al., 2017).

#### Acidity

Acidity is a major trait of grape berry quality driving the sensory properties of wines, their chemical and microbiological stability as well as aging potential. Grape acidity can be assessed by titratable acidity or pH. pH is determined by the content in organic acids, mainly malic acid and tartaric acid but also by cations, mainly potassium, that partly neutralize the organic acids (Boulton, 1980).

The genotypes used, both for scions and rootstocks varieties, play a major role in the final acidity of wines, with pH varying at harvest from 2.91 (Duchêne et al., 2014) to 4.36 (Kliewer et al., 1967) in *V. vinifera* grapes. Phenology is a confusing factor when trying to compare genotypes. Comparing acidity parameters for different genotypes, even after the same number of days after véraison, can be biased because malic acid degradation depends on temperatures during ripening (Duchêne et al., 2014). The tartaric acid concentration of the berries is far less sensitive to high temperatures than the malic acid concentration (Kliewer and Torres, 1972). Indeed, the quantity of tartaric acid per berry is generally considered constant throughout berry ripening (DeBolt et al., 2008). Grapevine varieties with a high tartaric/malic ratio should be better adapted to warmer climatic conditions. There is a genetic variability for the tartaric/malic ratio in grapevine genotypes (Shiraishi, 1995; Duchêne et al., 2014; Bigard et al., 2018). QTLs for pH and tartaric acid concentration have already been found in segregating populations (Viana et al., 2013; Chen et al., 2015; Houel et al., 2015; Ban et al., 2016; Duchene et al., 2020). Diversity panels were also studied to detect QTLs for the concentration of malic acid and tartaric acid (Liang et al., 2019) or for wine acidity (Laucou et al., 2018). These results open the gate for breeding varieties able to keep a correct level of acidity in the warm conditions of the future. The links between genetic variations in (Mal), (Tart), or (Mal)/(Tart) and genetic variations of pH have however never formally been established. The missing element is likely (K^+^). Indeed, Duchene et al. (2020) showed that malic acid concentrations, or the malic/tartaric acid ratios, were driven by strong QTLs on chromosomes 6 and 8, but were not associated with variations of pH. These variations of pH were explained by QTLs for the potassium-to-tartaric acid ratio, on chromosomes 10, 11, and 13.

(K^+^) in grape juices also depends on the rootstock used, which could induce variations of pH between 3.76 and 4.27 in “Shiraz” grapes (Kodur et al., 2013). Genetic variations for (K^+^) in leaves in hybrids from a rootstocks cross (Gong et al., 2014) open the possibility of breeding rootstocks for K^+^ accumulation in scions.

#### Aromas and Aroma Precursors

Empirical knowledge often associates wine quality with cool temperatures. Indirect results are showing that increasing temperatures are generally unfavorable to wine quality (Tonietto and Carbonneau, 1998; Tonietto and Carbonneau, 2004; Jones et al., 2005; Moriondo et al., 2010), but experimental data supporting this idea are rare. Correlations have been detected between temperatures and the concentrations of methoxypyrazines (Falcao et al., 2007) or C13-norisoprenoids, which result from the breakdown of carotenoids (Marais et al., 1992). Water stress can also modify the aromatic profiles of wines. 3-sulfanyl hexanol (3-SH) concentrations, for example, were significantly higher in Riesling wines when vines were irrigated (Pons et al., 2017). Many studies also highlighted the role of light exposure on the secondary metabolism in grape berries (Kwasniewski et al., 2010; Friedel et al., 2016). Shading grapes can however induce confusing effects between light and temperature (Bureau et al., 2000). Understanding the effects of temperature, light, and water availability on the synthesis of aromas and aroma precursors is a challenge for anticipating the effects of climate change and for proposing adaptation strategies.

Genetic approaches can show which genes are responsible for genetic variations.

Monoterpenols are 10-carbon molecules found in high concentration in berries of cultivars such as Gewurztraminer and varieties of the Muscat family. They are associated with floral aromas (Mateo and Jimenez, 2000). Duchene et al. (2009) and Battilana et al. (2011) demonstrated in three different progenies that a QTL for high terpenol synthesis colocalized with a gene coding for a 1-deoxy-D-xylulose 5-phosphate (DOXP) synthase; DOXP is the precursor of geranyl diphosphate (GPP), the substrate used by terpene synthases (VvTPS) to produce monoterpenols such as geraniol, linalool or α-terpineol. In aromatic genotypes, a mutation of a single base in the gene coding for the 1-deoxy-D-xylulose 5-phosphate synthase (DXS), is sufficient to enable a higher synthesis of DOXP, and further GPP, in aromatic cultivars (Battilana et al., 2011). These results were confirmed with genome wide association studies (Emanuelli et al., 2010; Laucou et al., 2018; Guo et al., 2019). A genetic approach also confirmed the role of a cytochrome P450 in the synthesis of carboxy-linalool, a precursor of wine-lactone (Ilc et al., 2017).

The pepper-like fragrance of methoxypyrazine is often not appreciated when concentrations are too high (Guillaumie et al., 2013). 2-methoxy-3-isobutylpyrazine (IBMP) is an example of methopyrazine, whose non-volatile precursor, 2-hydroxy-3-isobutylpyrazine, is methoxylated by an S-adenosyl-methionine-dependent O-methyltransferase, VvOMT3, to form IBMP. Guillaumie et al. (2013) detected a QTL for IBMP concentration in the progeny from a Cabernet-Sauvignon × Riparia Gloire cross that colocalized with VvOMT3. Variations of the level of expression of *VvOMT3* correlated with the level of IBMP synthesis.

Rotundone is the molecule responsible for the green peppery aroma in Shiraz grapes and wines (Siebert et al., 2008). Using a genomic approach, Drew et al. (2016) showed that variations at two amino acid positions in VvTPS24, a sesquiterpene synthase, were responsible for functional changes that allow the synthesis of α-guaiene, the precursor of rotundone. α-guaiene is then oxidized by the cytochrome P450 CYP71BE5 to form rotundone (Takase et al., 2016).

Knowing all the genes participating in aromas or aromas precursors synthesis is essential for more precise monitoring of mRNA synthesis according to environmental conditions or management practices.

#### Phenolic Compounds

Phenolic compounds are key components of wines: anthocyanins for berry color and condensed tannins for wine structure and astringency.

The decrease in anthocyanin content under high temperatures is well documented (Mori et al., 2007; Bonada et al., 2015; Lecourieux et al., 2017). Using empirical models linking berry composition and climatic data, Barnuud et al. (2014) forecasted a decrease of anthocyanins concentrations in the future for a given sugar level (22°Brix). Their simulation showed that this decrease could be higher for Cabernet-Sauvignon than for Syrah. Experimental data also showed that the loss of grape color under high temperatures was lower in Cabernet-Sauvignon or Pinot noir than in Tokay grapes (Kliewer and Torres, 1972). High temperatures do not reduce the concentrations of all anthocyanins with the same intensity: di-hydroxylated anthocyanins are more affected than tri-hydroxylated anthocyanins (Lecourieux et al., 2017), malvidin-3-*O*-glucoside less than delphinidin-3-*O*-glucoside (Lecourieux et al., 2017). A study combining a bi-parental cross and a core collection confirmed that a locus on chromosome 2 is responsible for berry color (Fournier-Level et al., 2009) and that, within colored varieties, genetic polymorphisms in the same genomic region are associated with continuous variations of anthocyanin concentrations (Fournier-Level et al., 2009). Data from Lecourieux et al. (2017) suggest that the effects of high temperatures are all the more significant as the number of methyl groups is lower. In parallel, Fournier-Level et al. (2011) detected a link between genetic variations on chromosomes 1 and 2 with the levels of anthocyanin methylation in a Syrah × Grenache progeny. They could associate two SNPs in a gene coding for an O-methyltransferase with the level of methylation. These results indicate that molecular markers can be used for breeding varieties with a high capacity to maintain their coloration under high temperatures. Costantini et al. (2015) also detected QTLs on 13 chromosomes that drive the anthocyanin profiles in a Syrah × Pinot noir progeny.

Quantitative trait loci from segregating populations or diversity panels were also proposed for proanthocyanidins synthesis (Huang et al., 2012, 2014; Carrier et al., 2013). These molecules are however less sensitive to temperatures than anthocyanins (Pastore et al., 2017) and are not critical in the challenge of adaptation to climate change.

## Controlling the Genome and Its Expression

Obtaining new genotypes with specific characteristics was for centuries performed by choosing plants showing new and interesting phenotypes among hundreds (mass selection). The next step was to cross two plants and to select the best individuals within a progeny. These methods relied on the observations of the phenotypes of the plants. Molecular tools allow now choosing plants according to genetic information at the DNA level. Modern technologies are also able to generate random mutations that are possibly interesting but the most promising one is the direct editing of the genome at a precise location.

### Breeding: Marker-Assisted Selection and Genomic Selection

The search for QTLs provides the breeder with statistical links between the presence of specific alleles at a given locus and the quantitative value of a trait. The strength of this relationship, the quantitative value of the variation due to allelic changes, the number of loci driving the trait of interest will determine whether the information can be used in breeding programs. For the grapevine, the generation of offspring from a bi-parental cross is time-consuming (manual castration and manual pollination). The number of genotypes in such progenies is often too small to allow selecting plants for traits depending on several loci with weak effects. In practice, MAS is only used for traits depending on a few loci with strong effects. This is the case for resistance to diseases (Merdinoglu et al., 2018), for berry color (Yang et al., 2016), or for the ability to produce terpenols at high concentrations (Emanuelli et al., 2014). The ability to characterize thousands of SNPs in a genome for a reasonable cost is the basis of the “Genomic selection” method (Meuwissen et al., 2001). Instead of trying to predict a phenotype with a few points in the genome identified by QTL detection, mathematical methods are used to take into account the genetic information of all the SNPs. Genomic selection is routinely used for dairy cattle selection at the industrial level (Wiggans et al., 2017). The general principle of genomic selection is to build genomic prediction models with a training population and use them to predict phenotypic traits in a breeding population with genetic information only, in order to choose the individuals combining the most interesting features. The interest of genomic selection for grapevine breeding was first evaluated by simulations (Fodor et al., 2014), and the best predictions were obtained by combining GWAS and genomic selection. Good prediction accuracy were only calculated when the breeding population was not too distant from the training population. Working with actual data, Migicovsky et al. (2017) calculated genomic prediction accuracies for 32 traits, reaching 0.76 for berry length. Genomic selection is expected to be more efficient than MAS for complex traits depending on many loci with small effects. New approaches based on artificial intelligence and neural networks are also underway (Gonzalez-Camacho et al., 2016).

### Creating Mutations

Targeting Induced Local Lesions in Genomes (TILLING) is a reverse genetics method that allows identification of mutations in genes of interest after inducing mutagenesis with a chemical mutagen. The following step is to establish links between mutations in a gene of interest and specific phenotypes to reveal the function of this gene (Henikoff et al., 2004).

Such an approach was attempted with the grapevine by the SVQV INRAE laboratory in Colmar using ethyl methanesulfonate (EMS) on the seeds collected on selfings of the PN40024 line, the nearly homozygous line that provided the grapevine reference genome (Jaillon et al., 2007). Several experiments led to the result that the sub-lethal EMS dose/treatment duration was 4 mM for 16 h. However, searching for mutations in 34 genes in 1,217 plants led to the conclusion that the number of mutations detected was too low to consider this population as a “tilling” population. Toxic effects of EMS certainly appeared before enough mutations were generated.

### Genetic Engineering

Transgenesis allows adding or modifying unique traits in cultivars without, in theory, modifying their desirable characteristics. Like in other economically important crops, the production of GM grapevine plants has attracted a lot of attention since the early 1990s. Historically, the first successful attempt to create GM grapevines was reported by Baribault et al. (1990) who used co-culture of shoot pieces with *Agrobacterium tumefaciens* to generate *in vitro* cultivated shoots expressing the GUS reporter gene. Severe limitations to this approach were noted, however: the obtained shoots consisted of a mosaic of wild-type and transgenic cells that failed to root and to regenerate plants. These issues were solved by the advent of embryogenic cell lines from various grape genotypes, which allowed regenerating “true” (non-mosaic) transgenic plant from single cells through somatic embryogenesis (Martinelli and Mandolino, 1994; Scorza et al., 1995; Mozsár et al., 1998). This paved the way to the obtention of the first generation of GMO grapevines, mostly tailored for pest resistance, by overexpressing defense-related genes. For example, the coding sequence of rice chitinase *RCC2* was introduced in the Japanese table grape Neo Muscat, under the control of the 35S promoter to breed resistance against *Uncinula necator* (Yamamoto et al., 2000). Coutos-Thévenot et al. (2001) transformed the rootstock 41B with a more elaborate construct bearing the grapevine stilbene synthase 1 *VST1* coding region under the control of the alfalfa, pathogen-inducible, PR10 promoter, conferring tolerance toward *Botrytis cinerea* to the transgenic plants. More recently, besides pest tolerance, new traits were gradually targeted for breeding through genetic transformation, including abiotic stress tolerance and fruit-related quality traits. Freezing tolerance was enhanced by overexpressing the cold-inducible *A. thaliana* Dehydration Response Element Binding (AtDREB1b) or the *V. Vinifera* C-Repeat Binding Protein 4 (VvCBF4) transcription factors in the table grape “Centennial Seedless” (Jin et al., 2008; Tillett et al., 2012). The aquaporin VvPIP2 was introduced in the cultivar “Brachetto” and expressed under the control of the 35S promoter by Perrone et al. (2012) in an attempt to produce grapevine plants more tolerant to drought stress. Finally, overexpression of the *VvMYBA1* master regulator in both red (Shiraz) and white (Chardonnay) cultivars, led to enhanced production of acylated anthocyanin, through transcriptional up-regulation of the anthocyanin acyltransferase *Vv3AT* (Rinaldo et al., 2015) paving the way to transgenic grape with improved fruit quality.

Even though the above-mentioned production of transgenic grapevine was technically successful, little, if any, made it to production vineyards, mostly because of both consumers and growers’ reluctance to accept transgenic grapes, on grounds of health and environmental concerns, at least in Europe (Fuchs, 2008). Next-generation plasmid-free CRISPR/Cas9 genome edition technique may have the potential to overcome this reluctance to accept GM grapes, or more generally crops (Malnoy et al., 2016). Recently, a genome-wide survey of suitable sites for CRISPR/Cas9 genome editing has been conducted in grapevine (Wang et al., 2016) and successful attempts to actually generate genome-edited grapevine have been reported (Ren et al., 2016; Wang et al., 2016). Although the latter were just merely proof of concept attempts, Wan et al. (2020) reported this technology to generate grapevine plants with enhanced powdery mildew resistance through *Mlo* gene edition. The authors reported a 38.5% successful gene edition rate, a value lower to those previously reported in rice (84.3% on average) but comparable to those obtained in Arabidopsis (35.6% on average) (Ma et al., 2015). The CRISPR/Cas9 technology was also used for creating plants expressing only one of the two main isoforms of the FLM gene involved in flowering regulation and was effective in producing early (FLM-δ expressing)- and late (FLM-β expressing)- flowering phenotypes (Figure 1; Capovilla et al., 2017). This demonstrates the crucial role of AS in determining phenological traits as well as the potentiality of genome editing for creating new varieties adapted to future climate change. Moreover, engineered CRISPR Artificial Splicing Factors have recently been shown effective for controlling AS in animal cell cultures, which constitutes a promising strategy to modify phenotypes by manipulating the transcriptome (Du et al., 2020). Thus, the technology has undoubtedly great potential for future grapevine, and more broadly plant breeding programs. Its actual use, however, will be largely dependent on local regulations. United States Department of Agriculture does not impose any GM restrictions on genome-edited plants if they are free of any foreign or transgenic DNA, thus there is a fair chance that CRISPR/Cas9 modified plant could be free of GM organism regulations, at least in the United States (Waltz, 2012; Jones, 2015). Conversely, in Europe and New Zealand, the current legal status of genome-edited plants classifies them as GM organisms, and the same regulations as for transgenic plants apply (Schmidt et al., 2020).

## Conclusion

Molecular tools for describing genome sequences, genetic variations among varieties or clones, levels of gene transcription, and protein quantification have evolved exponentially during the last decades. The first release of a reliable grapevine sequence in 2007 required several years of sequencing with the Sanger technology before attempting a puzzling assembly, whereas a complete sequence of a heterozygous variety, build with long reads of DNA, takes now only a few weeks. GBS technology allows now characterizing hundreds of genotypes at thousands of points in a genome in a single run of sequencing, and transcriptomic as well as proteomic tools follow the same trend. There is still a lot to learn on the regulation of gene transcription and AS, on the mechanisms of interfering RNAs, DNA methylation, or chromatin activity but also on the mechanisms regulating protein synthesis and turnover.

Adaptation of grapevine to new environmental conditions will be all the more efficient as the physiological responses to drought, elevated temperatures, or combined stress on plant growth, development, and berry composition are precisely described. To achieve this goal, the first challenge is to characterize the levels of stress imposed in experiments in a way the results can be extrapolated in other environmental conditions and that they make sense in real vineyard conditions. The second challenge is to develop and to use methods able to integrate and interpret large datasets that include genomic sequences, transcriptomic, proteomic, and metabolomic data. This requires huge efforts toward integrated network analysis and “system biology.” The final goal is to build a corpus of knowledge that includes the responses to quantified environmental variables and genetic variability.

Finally, this knowledge can help to construct adaptation strategies not only on the plant side for the control of gene expression, for breeding new varieties by hybridization or by genome editing technologies, but also on training systems and plant management techniques.

## Author Contributions

ÉD, ÉG, and PM carried out bibliographic searches, redacted the first draft on specific topics, and reviewed the whole manuscript. ÉD initiated and supervised the work. All authors contributed to the article and approved the submitted version.

## Conflict of Interest

The authors declare that the research was conducted in the absence of any commercial or financial relationships that could be construed as a potential conflict of interest.

## Footnotes

1 https://urgi.versailles.inra.fr/jbrowse/gmod_jbrowse/
